# Compassionate Use of Cefiderocol as Adjunctive Treatment of Native Aortic Valve Endocarditis Due to Extremely Drug-resistant *Pseudomonas aeruginosa*

**DOI:** 10.1093/cid/ciy963

**Published:** 2018-11-12

**Authors:** Jonathan D Edgeworth, Domenico Merante, Sanjay Patel, Christopher Young, Paul Jones, Seema Vithlani, Duncan Wyncoll, Peter Roberts, Andrew Jones, Tsutae Den Nagata, Mari Ariyasu, David M Livermore, Richard Beale

**Affiliations:** 1 Department of Infectious Diseases, Guy’s Hospital, Kings College London, London, United Kingdom; 2 Shionogi Limited, Global Clinical Development Unit, London, United Kingdom; 3 Intensive Care Unit, London Bridge Hospital, HCA International, London, United Kingdom; 4 Microbiology Department, HCA International, London, United Kingdom; 5 Pharmacy Department, London Bridge Hospital, HCA International, London, United Kingdom; 6 Shionogi & Co., Ltd., Kitaku, Osaka, Japan; 7 Norwich Medical School, University of East Anglia, United Kingdom; 8 Antimicrobial Resistance & Healthcare Associated Infections Reference Unit, Public Health England, Colindale, London; 9 School of Medicine, Guy’s Hospital, Kings College London, United Kingdom

**Keywords:** cefiderocol, endocarditis, *Pseudomonas aeruginosa*, drug resistance, microbial

## Abstract

Serious infections such as endocarditis due to extremely drug-resistance gram-negative bacteria are an increasing challenge. Here, we present successful adjunctive use of cefiderocol for a patient with persistently bacteremic healthcare-associated native aortic valve endocarditis due to an extended-spectrum beta-lactamase–positive *Pseudomonas aeruginosa* susceptible in vitro only to colistin, following failure of conventional therapeutic options.

Cefiderocol is a novel parenteral siderophore cephalosporin currently being developed to treat serious infections, including those due to carbapenem-resistant gram-negative strains [1–3]. Cefiderocol has a unique mechanism of cell entry via bacterial iron transport channels. This allows it to enter gram-negative bacteria efficiently, even when accumulation of other agents is reduced due to porin channel loss and increased expression of efflux pumps. Cefiderocol has good stability to all classes of beta-lactamases, including serine- or metallo-carbapenemases that hydrolyze most or all other beta-lactam antibiotics. This combination of properties allows potent antibacterial activity against a wide variety of Enterobacteriaceae and nonfermenting gram-negative bacteria including *Pseudomonas aeruginosa*, *Acinetobacter baumannii*, and *Stenotrophomonas maltophilia*, even when these have a potent combination of beta-lactamases. Activity is poor against gram-positive bacteria and anaerobic bacteria [1, 3].

Cefiderocol has been assessed in early-phase clinical trials for safety, tolerability, and pharmacodynamic behavior [4, 5]. Cefiderocol was shown to be safe and effective for the treatment of complicated urinary tract infection in a recently completed phase 2 study (APEKS-cUTI study) involving 452 randomized patients [6]; it is now being evaluated in phase 3 studies (CREDIBLE-CR and APEKS-NP). Shionogi, the developers of cefiderocol, will consider unsolicited requests for compassionate use and were approached to assist with treatment in the following case.

A 78-year-old woman was admitted from an intensive care unit in Kuwait to London Bridge Hospital on 30 October 2017 for specialist urological and respiratory management. She had been in hospital in Kuwait for 3 months following complications of hydronephrosis secondary to a spontaneous ureteric hematoma. She had a past medical history of aortic stenosis, ischemic heart disease, and cerebral infarction and was in remission from breast cancer.

On arrival in London, she was ventilated and received intermittent hemofiltration without hemodynamic support or antimicrobials. Admission blood cultures grew extremely drug-resistant (XDR) *P. aeruginosa* susceptible to gentamicin, amikacin, and colistin (minimum inhibitory concentration [MIC] <3 mg/L) but resistant to all beta-lactams and quinolones. There was no in vitro synergy between antipseudomonal agents and fosfomycin or rifampicin. The isolate lacked carbapenemase genes, and resistance to these agents was inferred to reflect loss of porin (OprD). A *bla*_(Vietnam ESBL)_ gene was found by polymerase chain reaction (PCR) assay and was considered to account for resistance to penicillins and cephalosporins, including ceftazidime/avibactam and ceftolozane/tazobactam. The patient also had rectal colonization with OXA-48 *Klebsiella pneumoniae* and OXA-23/OXA-51 *A. baumannii*. She was commenced empirically on colistin (9 megaunits (MU) loading dose followed by 3 MU 3 times per day, subsequently changed to 4.5 MU 2 times per day) together with intermittent gentamicin based on serum levels. A transthoracic echocardiogram showed a thickened aortic valve but no obvious regurgitation or vegetation. Representative clinical and microbiological features from her admission are presented in Figure 1.

**Figure 1.**
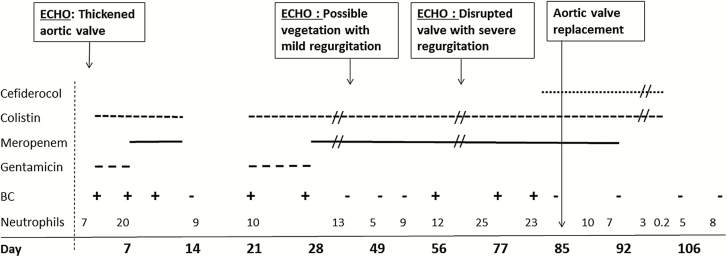
Clinical, microbiological, and antimicrobial treatment course. Abbreviations: BC, blood culture positive (+) *Pseudomonas aeruginosa* or negative (−); ECHO, transthoracic echocardiogram.

She became clinically septic following insertion of percutaneous nephrostomies and started high-flow continuous veno-venous hemofiltration (estimated creatinine clearance 40–50 mL/minute). Blood cultures again grew *P. aeruginosa* on days 3, 7, and 10. Gentamicin was stopped after the third dose on day 5 and replaced with meropenem 2 g 2 times per day, although MICs of the drug for all the *P. aeruginosa* isolates were >32 mg/L. Sepsis resolved on day 14, with a negative blood culture and C-reactive protein (CRP) falling from 212 to 41; this improvement prompted cessation of antibiotic therapy. Sepsis returned within a few days, however, and the CRP rose to 267 mg/L, with day 22 and 27 blood cultures again growing *P. aeruginosa*, leading to recommencement of colistin and gentamicin. CRP continued to rise to 327 mg/L, and a day 27 *P. aeruginosa* isolate was reported as intermediately resistant to gentamicin, so gentamicin was again switched to meropenem. A second transthoracic echocardiogram showed possible vegetation on a tricuspid aortic valve with mild to moderate regurgitation.

Blood cultures were negative on days 38, 49, and 52, and the patient initially improved clinically while undergoing assessment for aortic valve surgery. However, during that time, there was a significant neurological deterioration with a computed tomography scan showing multifocal infarcts consistent with embolization; blood cultures again became positive (days 56, 62, and 68). A decision was made to source additional active antibiotics to control bacteremia prior to valve surgery.

## ADJUNCTIVE TREATMENT WITH CEFIDEROCOL

Accordingly, a formal request was made to Shionogi on day 73 for compassionate use of cefiderocol. The request was granted, and the drug was obtained after appropriate governance approvals. Disk diffusion testing performed on day 3 and day 68 isolates gave zones of 17.4 and 21.3 mm, respectively, against a prospective 18-mm breakpoint for a 30-μg disk. Cefiderocol was administered from day 83 while continuing meropenem and colistin, and aortic valve replacement was performed on day 85. Intensivists decided on a cefiderocol dose of 2 g administered over 3 hours 3 times a day for the first 2 days then 2 g twice daily, based on a combination of renal function, septic state, complexity of the infection, and laboratory susceptibility testing. Blood culture taken before the first cefiderocol dose remained positive, but a blood culture taken after the sixth dose, on the day before surgery, was negative after 5 days of culture. The valve appeared heavily infected and disrupted at surgery and was positive for *P. aeruginosa* by PCR. Nevertheless, no gram-negative bacteria were seen on microscopy, and no growth was obtained, including on enrichment culture. Meropenem was stopped a week after surgery, but the cefiderocol/colistin combination was continued for an additional 3 weeks. The neutrophil count fell during the last 4 days of antibiotic treatment to a low of 0.2 10^9^/L on the planned last day of antibiotics, then returned to the normal range within a few days after stopping antibiotics. Neutropenia was reported in the serious adverse event report form, with either colistin or cefiderocol being considered the most likely potential causes. Multiple blood cultures after surgery and after stopping antibiotics were negative up to day 275 while the patient received convalescent care with slow neurological recovery leading to transfer back to Kuwait.

## DISCUSSION

Infective endocarditis due to *P. aeruginosa* is rare, except among intravenous drug users, accounting for <0.5% of all endocarditis cases [7]. A recent literature review of 27 cases over 20 years reported 75% as healthcare associated, of which half required surgery and a third relapsed after apparent adequate treatment [8]. *Pseudomonas aeruginosa* endocarditis is usually treated with combination therapy, such as meropenem or ceftazidime plus an aminoglycoside [7], but these regimens are compromised against XDR strains, as in this case. Colistin is usually used as backbone therapy for XDR gram-negative bacterial infections, often with meropenem. However, in this case, a combination of these agents achieved only temporary blood culture sterility, with no other identified available options. Antibiotic dosing was at the high end of the recommended range, and a trough colistin serum level was measured at 6.3 mg/mL (target 2–4 mg/mL) 2 weeks prior to surgery, so it was considered that adequate doses were used.

The cause of the endocarditis was not identified but was probably present prior to transfer from Kuwait. *Pseudomonas aeruginosa* was not grown from any other cultures, including samples from the urinary tract. There were no predisposing factors such as a prosthetic valve or the presence of pacing wires, as reported in other studies [9]. Although *P. aeruginosa* endocarditis can be treated with antibiotics alone, the failure to achieve sterility in this case combined with evolving valvular destruction meant surgery was essential for any prospect of cure. However, this was delayed in part due to embolization and was considered unlikely to succeed with persistently positive blood cultures on antibiotic therapy. The addition of cefiderocol led to blood culture sterility within 2 days, allowing potentially curative surgery to proceed. It is not possible to ascribe the relative contribution of surgery vs cefiderocol, but the rapid blood and valve sterility after starting cefiderocol suggest that this cephalosporin made a significant contribution. This report adds to the evidence from ongoing randomized studies that cefiderocol holds promise of being a useful new agent for treating XDR gram-negative bacteria, including when no other options exist.

From a microbiological perspective, it is striking that for *P. aeruginosa*, where there is easy mutational resistance to carbapenems, an extended-spectrum beta-lactamase (ESBL) gave the broadest spectrum of resistance. Also striking, and typical of VEB ESBLs in *P. aeruginosa*, is the fact that resistance included ceftolozane/tazobactam and ceftazidime/avibactam [10]. Although VEB enzymes are uncommon in the United Kingdom (and the United States), the number of producer is increasing, often via imports from the Middle East and Eastern Europe [10], where these enzymes appear to be more prevalent.

## CONCLUSIONS

The emergence of multiresistant and extremely resistant gram-negative pathogens presents a global health challenge and underscores the urgent need for new antibiotics [11, 12]. Cefiderocol is being developed to address this need, targeting Enterobacteriaceae, *P. aeruginosa*, *A. baumannii*, and *S. maltophilia* [1, 2].

This case report documents a patient with XDR *P. aeruginosa* who was successfully managed with the addition of cefiderocol to control bacteremia and to allow aortic valve replacement. Compassionate use of cefiderocol was opted for this patient because there were no other available therapeutic options and because conventional agents were failing to control persistent bacteremia. An episode of transient acute neutropenia occurred during the last few days of treatment with both cefiderocol and colistin, highlighting the importance of continued pharmacovigilance during extended courses of antibiotics.

## Notes

***Acknowledgments.*** We are grateful to Rachel Pike and Daniele Meunier at Public Health England (PHE) for susceptibility testing and molecular characterization of the isolates. We are also grateful to Hamish Rankin at Shionogi Limited London UK for the clinical operations activities that led to the provision of cefiderocol.

***Financial support.*** PHE is funded by the UK government.

***Potential conflicts of interest.*** J. D. E. served on advisory boards for Pfizer and Merck. D. W. reports personal fees from Merck, Pfizer, Johnson & Johnson, and Fisher & Paykel. D. M. L. has served on advisory boards or ad-hoc consultancy for Accelerate, Achaogen, Adenium, Allecra, Basilea, BioVersys, Centauri, Integra Holdings, Meiji, Melinta, Nordic Pharma, Pfizer, F Hoffmann-la-Roche, Shionogi, Taxis, T.A.Z., Tetraphase, the Medicines Company, VenatoRx, Wockhardt, and Zambon; has received personal fees for paid lectures from Astellas, AstraZeneca, bioMérieux, Beckmann Coulter, Cardiome/Correvio, Cepheid, Merck, Pfizer, and Nordic; has received research funding from Melinta, Merck, Paratek, Roche, and VenatoRx outside the submitted work; has performed contract research for Merck, Melinta, Pfizer, and Wockhardt; and has relevant shareholdings with Dechra, GlaxoSmithKline, Merck, Perkin Elmer, and Pfizer amounting to <10% of portfolio value. D. M. L.’s affiliation, PHE, is funded by the UK government and has undertaken contract research on cefiderocol for Shionogi. D. M., S. P., T. D. N, and M. A. are employees of Shionogi Ltd. All other authors: No reported conflicts. All authors have submitted the ICMJE Form for Disclosure of Potential Conflicts of Interest. Conflicts that the editors consider relevant to the content of the manuscript have been disclosed.
